# Round the Hospitals

**Published:** 1916-04-29

**Authors:** 


### THE MATRONS' AND SISTERS' DEPARTMENT.

### ROUND THE HOSPITALS.

We have the pleasure to call attention to the important letter of the Hon. Arthur Stanley, the chairman of the Council of the College of Nursing, which appears on page 107. It is explanatory and of supreme importance. It is calculated to remove several misapprehensions. It demonstrates the all-embracing character of the scheme, its self-government, its democratic basis, and how it is proposed to secure the solidarity of the nursing profession as a matter of paramount importance. The scheme is to include the whole of the United Kingdom, whilst the constitution, functions, and work of the Consultative Board are fully explained. The hope is expressed that no time will be lost by the boards of the hospitals, infirmaries, and nurse-training schools throughout the country each to nominate not more than two representatives upon the Consultative Board of the College within the next month. A careful study of this manifesto should convince all impartial and knowledgeable persons throughout the nursing world that, the College of Nursing deserves whole-hearted support, for the scheme it represents is capable of uniting the whole nursing profession and of giving to it a position which cannot fail to claim and receive State recognition at the earliest practicable moment.

---

The letter of the Chairman of the College of Nursing will remind those responsible for the training and examination of nurses throughout the country of the importance of putting forward at the present time points in their school to which they properly attach special importance or value. We hope next week to give some particulars of the plan pursued in a provincial training school of importance, with examples of its examination questions, together with the methods of marking pursued by each lecturer attached to the school. We invite further contributions of the same kind, which should be sent to the Editor as quickly as possible, so that the information given may be made available through The Hospital for the consideration of those interested, whilst these questions are exciting the keenest interest.

---

The following Liverpool notes have been kindly sent by the matron. "I was interested in 'Easter Day, 1916,' in The Hospital of last week. As you know, we have a beautiful chapel here, which was greatly appreciated this Easter and during Holy Week. There were more than 130 communicants on Easter Day, the 6 a.m. Celebration being attended by the nurses and some maids, as well as by a number of convalescent soldiers, who were allowed to attend with us instead of later. Sixty patients received Communion in bed; the chaplain and his two curates attended in each ward, the sisters having previously provided me with a list of their patients who wished to communicate, with the number of each bed. We have had a weekday service during Lent, which has been well attended by the soldiers (quite spontaneously), who came on Sunday mornings in force and sang most lustily, which was a real enjoyment to the rest of us. I was also impressed by a number asking permission to attend the Celebration on Thursday last, which I was only too glad to give, as I think the privilege is ours in having our own chapel with its services for those of our men who care to attend." We shall be glad to have from the matrons of other hospitals their experiences too.

---

There is no doubt that among the changes and developments that await the nurse's training, her uniform will undergo modification. The tendency to replace the bonnet and cloak with the more sensible hat and overcoat will herald other changes in nurses' clothing. The overall has undoubtedly come to stay, and will be increasingly used while the nurse is in attendance on her patients. Frills and bows will tend to disappear, but perhaps the most-to-be-desired change is the abolition of that instrument of torture, the stand-up collar, which cuts the nurse's neck, wears out her dresses, and whose washing and polishing demand skilled labour and expensive machinery. It is to be hoped that the nurse of the future will wear a soft white piqué collar and cuffs. The former when fitting closely to the back of the neck, but open in front, is tidy, smart, and becoming. If attached to the dress by studs it is easily adjustable, and is—most important of all—extremely comfortable. Many matrons may encourage this reform in nurses' uniform collars.

At this time, when the need for economy claims the attention of hospital authorities in a special degree, there is some danger that the rising price of food may be the cause of leading matrons in their anxiety to keep down expense into what may be called extravagant economies. Undoubtedly a good deal of pressure will have to be, and must be, resisted if matrons are to prevent any reduction in the quality and quantity of food provided for patients, nurses, and servants. In well-managed hospitals matrons had brought the commissariat to an irreducible minimum before the outbreak of war. In such cases the only economies must take the direction of prevention of waste, not of reduction of quantity or quality of food for patients who need extra nourishment to replace losses. Similarly, nurses and others leading active lives actually require more food than women who lead a sedentary life. It is greatly to be hoped that cakes, milk, eggs, jam, cocoa, and so forth, will, in spite of their increased cost, continue to be regarded as necessities which cannot be ruled out of the hospital food bill.

---

The Walsall Board of Guardians have received an application from a blind girl who desired employment as a probationer nurse, and there seems to have been some discussion before the decision not to entertain the application was arrived at. It is difficult to imagine in what capacity a person deprived of sight could possibly be employed in a hospital other than as a masseuse. Possibly an application to an Institute for the Blind might lead to her receiving instruction in this branch of nursing, for a girl who has the pluck to wish to become a nurse in spite of the great disability under which her blindness would place her, should be worth training as a masseuse.

---

Another instance of the cheering effects upon hospital life of warrior patients comes from the South Devon and East Cornwall Hospital, Plymouth, where a piano purchased with the proceeds of a concert given by the Plymouth Madrigal Society was presented to the Lopes Ward on the 17th instant. This is the second piano presented to the hospital since the beginning of the war, for the entertainment of the wounded. At Plymouth we find not only musical instruments, but trained musicians and vocalists combined in generous harmony to cheer the inmates of the hospital. The members of the Madrigal Society attended the presentation and gave many delightful selections from their repertoire, the new piano being used. Not only so, but the numbers included a choir whose singing was much appreciated, whilst violin solos and recitations were comprised in the programme. Those present included fifty sick and wounded sailors and soldiers, who formed an appreciative audience, and supplied an eloquent speaker, in the person of Sergeant-Major Currie of the Irish Rifles, to express grateful thanks on behalf of his comrades. We hope that many Madrigal

**MATRONS' AND SISTERS' DEPARTMENT**—(*continued*).

Societies and other concert parties throughout the country may accept and follow the lead Plymouth has given. Then, through some of their members, every season's programme of each Madrigal Society for the future will include a hospital night or nights to brighten the days of the suffering patients in their midst.

---

The outlook for the matron, her responsibilities and difficulties, are usually greater than those of any member of the nursing staff, but an able sister of experience and zeal may sometimes press matron hard in these respects. We hope that each and both through the great developments of the present time may be quickened to put on their thinking-cap, to weigh their experiences, and to jot down such of them as may be calculated to prove interesting and helpful to other workers. One way to make the College of Nursing rapidly efficient is by the accumulated practical knowledge which our readers might thus supply to the Editor of The Hospital, who will gladly co-operate to the utmost to make both thoughts and experiences of immediate practical value.

---

Our readers may have noticed that we have been giving week by week a story in this section. These stories have come from old sisters, matrons, and others. There must be a large number of excellent stories current throughout the hospital world, which not only include Royal personages, but the patients, workers, and departments in the field which every hospital covers. Each story should be terse and possess an element of brightness. It may include some clear point, with humour, pathos, or suggestion. To encourage our readers we have decided to offer *a special prize of half a guinea* to the reader who sends to the Editor the best story he receives every month, in addition to the usual fee for each contribution when published. A sure test of excellence may be taken to be: If the story gives matron pleasure it will give our readers pleasure too.

---

"Hurrah!" Then the small patient became suddenly silent as the first of the guests stopped at the foot of his cot. "Getting better, little man?" Instead of replying, the child muttered "He can't be the Queen's son, he've took off his hat to we." But it was the Queen's son, the Prince of Wales, afterwards Edward VII., who had come with Alexandra Princess of Wales and two young daughters to a London hospital. When a novel kind of splint attracted the young Princesses, it was their father who asked if the patient complained of the confined position, but the child's contented smile reassured the Prince. Each child had a kindly recognition, as well as a rose from the Princess's bouquet. On the departure of the Royal visitors all the children agreed with a boy who exclaimed, "They're a jolly sort, them two, and chance it!"

---

⁂ It is our invariable practice to pay for all items of news which we may publish. The minimum payment is 5s. Paragraphs of about twenty lines in length—that is to say, of 150 words or less—are preferred. In this section during the war we propose to give 10s. to the correspondent who may send us in any week the best incident connected with hospital life and work. In this way we hope to afford to all practical aid and encouragement; while those who reciprocate will be able to render us invaluable service. Contributions should be addressed to The Editor, The Hospital, 28 and 29 Southampton Street, Strand, London, W.C., and, to be in time for the current week's issue, should reach him by the first post on Mondays.

